# The Role of Cities in Reducing Smoking in China

**DOI:** 10.3390/ijerph111010062

**Published:** 2014-09-26

**Authors:** Pamela Redmon, Jeffrey Koplan, Michael Eriksen, Shuyang Li, Wang Kean

**Affiliations:** 1 School of Public Health, Georgia State University, P.O. Box 3995, GA 30302, USA; E-Mail: meriksen@gsu.edu; 2 Global Health Institute, Emory University, 1599 Clifton Road NE, GA 30322, USA; E-Mails: jkoplan@emory.edu (J.K.); shuyang.li@emory.edu (S.L.); 3 ThinkTank Research Center for Health Development, Tian Bao Yuan Liu Li, Beijing 100176, China; E-Mail: wka2006@sina.com

**Keywords:** tobacco control, social norms, smoke-free policy, public health, capacity building

## Abstract

China is the epicenter of the global tobacco epidemic. China grows more tobacco, produces more cigarettes, makes more profits from tobacco and has more smokers than any other nation in the world. Approximately one million smokers in China die annually from diseases caused by smoking, and this estimate is expected to reach over two million by 2020. China cities have a unique opportunity and role to play in leading the tobacco control charge from the “bottom up”. The Emory Global Health Institute—China Tobacco Control Partnership supported 17 cities to establish tobacco control programs aimed at changing social norms for tobacco use. Program assessments showed the Tobacco Free Cities grantees’ progress in establishing tobacco control policies and raising public awareness through policies, programs and education activities have varied from modest to substantial. Lessons learned included the need for training and tailored technical support to build staff capacity and the importance of government and organizational support for tobacco control. Tobacco control, particularly in China, is complex, but the potential for significant public health impact is unparalleled. Cities have a critical role to play in changing social norms of tobacco use, and may be the driving force for social norm change related to tobacco use in China.

## 1. Background and Introduction

China is the epicenter of the global tobacco epidemic. Production and consumption of tobacco is the highest in the world, 43% of the world’s tobacco is produced in China, and its population smokes 38% of the cigarettes produced worldwide [1]. Fifty-three percent of Chinese men smoke, as do 2.4% of women. The total smoking population is estimated to be greater than 300,000,000 [2].

Approximately one million smokers in China die annually from diseases caused by smoking, and this estimate is expected to reach over two million by 2020 [3]. Non-smokers exposed to secondhand smoke, particularly women, are also at increased risk of death and disease. In fact, it is estimated that more women die from exposure to secondhand smoke than die from smoking cigarettes themselves [3]. Seven out of ten non-smoking adults are exposed weekly to secondhand smoke [4].

China, the world’s largest producer of cigarettes, manufactured 2.29 trillion cigarettes in 2009 [5]. Because China’s tobacco industry is state-owned, the central government benefits greatly from the revenue—both excise tax revenue and profit. In 2007, the Chinese government profited 388 billion RMB from the sale of cigarettes, approximately 7.56% of the entire central government revenue [6].

Curbing the tobacco epidemic in China will require the implementation of effective tobacco control measures. Despite the financial benefits the Chinese government derives from the sale of tobacco products, government leaders are beginning to consider the long-term costs of the tobacco epidemic and the need to change the *status quo*. In recent years, the central government has begun to engage in limited tobacco control efforts. In 2006 China ratified the WHO Framework Convention on Tobacco Control (FCTC), with a commitment to implement the obligations contained in FCTC [7]. The Ministry of Health issued a smoke free hospital policy in 2009, with guidelines for healthcare facilities to be smoke-free by 2011 [8], and the Ministry of Health and the Ministry of Education also issued a policy with guidelines for creating smoke free schools [9]. The Ministry of Health authorized a policy prohibiting smoking in indoor public places starting 1 May 2011 [10], and the National People’s Congress included a statement of commitment to tobacco control in the 12th 5-year Plan [11]. Unfortunately, assessments of the implementation of the FCTC show many gaps in fulfilling that obligation [12].

While “top down” measures are important and necessary for fulfilling the obligations of the FCTC, successful tobacco control efforts require acceptance and enforcement at the local level. China cities have a unique opportunity in leading the tobacco control charge from the “bottom up”. Cities have the authority to pass and enforce smoke free policies and can change social norms through targeted public health education efforts and media campaigns. Cities can also adopt policies restricting advertising and corporate sponsorship. The enforcement of national polices occurs at the local level. And compliance with policies and enforcement of regulations requires a population that accepts such control measures, if not even demands them.

Given that there are over 160 cities in China each with a population exceeding one million residents, cities are a logical and strategic focus for intervention. Cities in China have the important role of implementing and enforcing central government policies, and they also have the ability to adopt stricter policies through Mayor’s directives or legislated, city-level policies. City-level public health organizations in China, such as the municipal Health Bureau, the municipal Centers for Disease Control, and the Patriotic Health Campaign Committee, have the responsibility of protecting the public’s health by promoting health education, preventing and controlling diseases, and implementing and enforcing health related laws, regulations, and policies. Cities have a unique opportunity to not only lead the efforts to educate their citizens and protect them from the harms of tobacco use and secondhand smoke, but also to take responsibility for implementing and enforcing central government guidelines and policy, increasing the likelihood that national priorities will actually be successfully implemented at the local level.

Article 8 of the FCTC calls for measures to adopt and promote effective policies to protect the non-smoker from secondhand smoke [6]. In 2008, it was reported that approximately one-half of cities in China have smoke free policies, but most of them remain noncompliant with Article 8 [13,14,15]. In recent years, some cities such as Guangzhou, Tianjin and Harbin have taken important steps towards fulfilling the requirement outlined in Article 8 and have adopted strict smoke free public place policies [16,17,18].

A city-based, “bottom up” approach to tobacco control in China is not a new concept. In 1999, the World Bank funded the Health VII Project. Health VII provided funds to seven China cities to establish public health projects, and one of the areas of focus was tobacco control [19]. In 2003, the Johns Hopkins School of Public Health received a Fogarty International Center grant to provide funding to cities to improve tobacco control capacity and to implement interventions in cities and rural areas [20]. The Bloomberg Philanthropies and the Bill & Melinda Gates Foundation have also provided significant funding for city-based tobacco control efforts since 2007 [21,22].

The Emory Global Health Institute was awarded a grant in 2008 from the Bill & Melinda Gates Foundation to support the development of effective, accountable, and sustainable tobacco prevention and control initiatives in China. The Emory Global Health Institute—China Tobacco Control Partnership (GHI-CTP) was established, and a major focus of the program is to change social norms of tobacco use through a “bottom up” approach in China cities. The GHI-CTP Tobacco Free Cities grant program (TFC) provides funds and delivers targeted training and tailored technical support to 17 selected cities to establish tobacco control programs, with specific smoke free policy, targeted programs and media and health education interventions selected by the cities themselves, aimed at changing the social norms of tobacco use.

The TFC program was modeled after the Health VII Project of the World Bank (1999–2006) that showed promising results in efforts to curb tobacco use and associated disease burdens including adoption of tobacco control policies, increased knowledge on negative effects of tobacco use, and implementation and utilization of cessation services [19,23]. The project evaluation concluded that policy change can occur, social norm change can be achieved, and surveillance systems can be implemented at the city level in China. It also confirmed that cities were manageable geographically and that city mayors in China have political power to make policy decisions related to tobacco [17]. Implicit in the Health VII Project experience was that when cities set their own health priorities, they were most likely to be successful.

The lessons learned from Health VII that were applied to developing the model for the TFC initiative included acknowledging the important role of government in promoting public health; ensuring that intervention strategies were grounded in science, applied to appropriate targets and had an expansive reach; ensuring adequate capacity of technical teams by providing needed training and technical support; conducting landscape analyses to better understand the tobacco control situation; and requiring surveillance and evaluation strategies to monitor effectiveness of the program [19].

**Figure 1:**
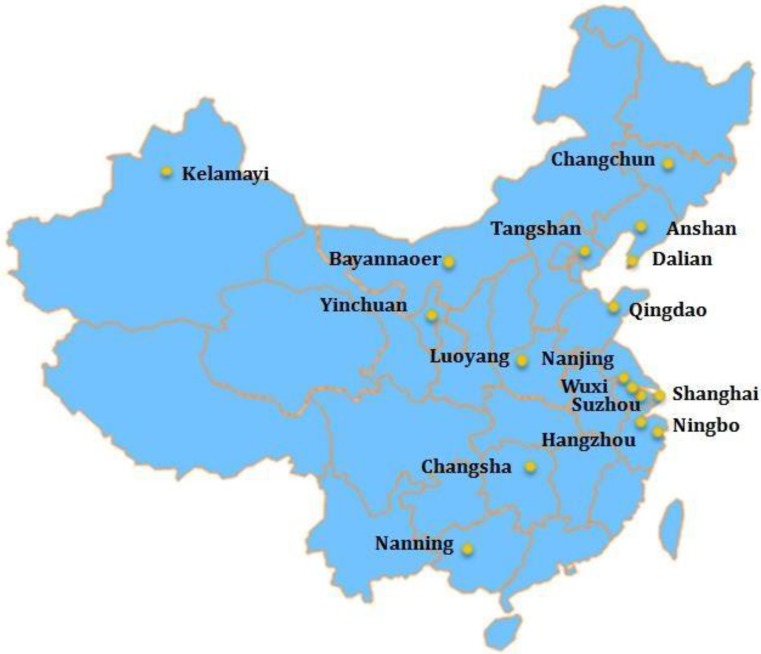
Emory GHI—Tobacco Free Cities.

The GHI-CTP collaborated with the ThinkTank Research Center for Health Development (ThinkTank, Beijing, China) to identify a non-random sample of potential cities for the TFC program. Site visits and telephone interviews were conducted to determine interest and level of commitment to the program goals. Seventeen cities were enrolled in the TFC program, and were selected based on size, geographic location, political will of the city leadership, especially the mayor, support of several leading public health units of the city, characteristics of the industrial, economic and social structure of the city and expressed enthusiasm to address the challenge of tobacco control. The 17 cities were also selected because they seemed most conducive to tobacco control and likely to be considered “innovators” or “early adopters” as described by the Diffusion of Innovation Theory [24]. Seven cities were included in the pilot program that began in July 2009—Ningbo, Wuxi, Luoyang, Tangshan, Shanghai, Qingdao, and Changsha. An additional ten cities were added to the program in July 2010—Anshan, Kelamayi, Bayannouer, Yinchuan, Changchun, Dalian, Hangzhou, Suzhou, Nanjing, and Nanning (Figure 1).

The population varies widely among cities, from 330,000 in Kelamayi to 19.2 million in Shanghai. Ten of the cities have tobacco industry presence—local crop growing, cigarette factories and/or company offices. The city organizations receiving grant funds to implement the TFC program include Patriotic Health Campaign Committees [3], Public Health Bureaus [5], Centers for Disease Control [5], and the Health Education Institute [4] (Table 1). These municipal organizations were selected because they have the responsibility to enforce the health policies, promote health and monitor health-related indicators, and educate the public on health-related issues.

The 17 TFC grantees were provided training opportunities, technical assistance, and grant funds to develop, implement, and evaluate strategies aimed at changing the social norm of tobacco use in their cities. The TFC grantees were required to select tobacco control strategies based on their unique situation and to draft an evaluation plan to assess the impact of their program. The TFC program model was designed to acknowledge the expertise and experience of the grantee as well as the unique situation of the city rather than utilizing a “one-size-fits-all” model of tobacco control.

The TFC grantees conducted a systematic city-wide situational analysis to better understand the extent of the tobacco use problem, existing tobacco control efforts, and to identify potential partners. The grantees selected the program goals based on their perceived ability to achieve the greatest initial impact and the potential for social norm change. The GHI-CTP required that the grantees’ plans include policy, program, and media and health communication components, and were reviewed and approved by the GHI-CTP team.

The TFC grantees targeted sectors for policy efforts such as hospitals and community clinics, families, government agencies, schools, enterprises, restaurants, hotels, and religious sites. Seven grantees included a legislated smoke-free public place policy component in their plans, with the goal of achieving a stronger policy than the existing central government policy. The plans also included targeted programs to support the policies, and strategies to engage the news media to inform and educate the public and mass media campaigns. The TFC grantees were required to develop and implement a process and outcome evaluation plan for their program. Each evaluation plan was tailored for the unique city program, and templates for evaluation instruments addressing the same sector or population were provided to the cities to allow for consistency for comparisons between programs.

The purpose of this paper is to demonstrate the role of cities in tobacco control and changing social norms of tobacco use in China. It will also identify facilitators and barriers to successful subnational tobacco control efforts.

## 2. Methods

The results are based on qualitative analysis of the bi-annual progress reports, progress on self-determined goals, objectives and activities outlined in the grantees’ action plans, policy documents, policy inspection reports, and media mentions in various media outlets.

### 2.1. Participants

Table 1 provides a listing of the 17 cities that participated in the TFC program. It also includes the funding organization.

**Table 1:** Emory GHI—Tobacco Free Cities grantees.

City, (Population, GDP in Billion Yuan)	Funded Organization		Progress Status		Smoke-Free Policy Goal (Adoption Date or *n*)			Policy Inspection Frequency		# of Media Mentions	Mass Media Campaign	
Anshan (3,503,584, 262.87) [25]	Health Education Institute of Anshan City		3		• SF public places (September 2012); effective January 2013			Quarterly		38		
					• SF Angang Steel Corporation (March 2012)							
Bayannaoer (1,669,000, 81.33) [26]	Bayannaoer Center for Disease Control and Prevention		1		• SF schools (October 2011)			Monthly		8		
Changchun * (7,569,000, 445.66) [27]	Changchun City Health Education Center		3		• SF schools (July 2011)			Monthly		50		
					• SF government worksites (July 2011)							
Changsha * (7,146,600, 639.99) [28]	Patriotic Health Committee of Changsha		2		• SF homes and families (*n* = 1057)			Quarterly		75	√	
					• SF hospitals (July 2011)							
Dalian (5,903,000, 700.28) [29]	Dalian Patriotic Health Campaign Committee		2		• SF schools (May 2012)			Quarterly		16		
Hangzhou * (5,477,000, 3460.6) [30]	Hangzhou Center for Disease Control and Prevention		3		• SF hotels & restaurants (September 2011)			Monthly		300	√	
Kelamayi (375,789, 81.0) [31]	Health Education Institute of Kelamayi		3		• SF public places (November 2012); effective March 2013			Quarterly		14	√	
					• SF government agency (March 2011)							
					• SF enterprise—Xinjiang Dushanzi Tianli High & New Tech Co Ltd. (March 2011)							
Luoyang * (6,885,400, 300.11) [32]	Luoyang Public Health Bureau		2		• SF homes and families (*n* = 62,538)			N/A		18		
Nanjing * (6,384,800, 720.157) [33]	Nanjing Office of Patriotic Health Campaign Committee		2		• SF primary and junior high schools (April 2011)			Once every 4 month		60	√	
Nanning * (7,135,000, 250.355) [34]	Nanning Health Bureau		2		• SF government agency (February 2012)			Twice per year		124	√	
					• SF China-ASEAN Expo and International Folk Song Festival (May 2012)							
Ningbo * (5,777,000, 652.47) [35]	Ningbo Center for Disease Control and Prevention		1		• SF primary and middle schools (June 2012)			Twice per year		20	√
Qingdao * (7,695,600, 730.211) [36]	Qingdao Center for Disease Control and Prevention		3		• SF hospitals (2009)			Twice quarterly		78	√
Shanghai * (23,804,300, 2010.133) [37]	Shanghai Municipal Health Bureau		1		• SF homes and families (*n* = 3083)			N/A		6	√
Suzhou * (6,478,100, 1201.165) [38]	Suzhou Center for Disease Control and Prevention		2		• SF government agency (April 2011)			Monthly		4	√
Tangshan (7,417,800, 586.163) [39]	Patriotic Health Campaign Committee		2		• SF government agency (May 2010)			Weekly		127	√
Wuxi (4,700,700, 756.815) [40]	Wuxi Health Bureau		2		• SF hospitals (March 2010)			Once every 2 month		142	√
Yinchuan (2,046,300, 114.083) [41]	Yinchuan Institute of Health Education		1		• SF government agency (July 2011)			Monthly		20	
					• SF religious sites (May 2011)						

Notes: * Tobacco industry presence. Progress Status Key: 1 = made progress, 2 = met goals, 3 = exceeded goal expectations.

### 2.2. Data Collection Procedures

TFC grantees were required to complete bi-annual progress reports. The progress report template included progress to date based on their action plans, facilitators and barriers to success, and lessons learned. Site visits and monthly conference calls with the grantees were used to validate the report information. Smoke-free policies and policy inspection were collected, and media mentions in various media outlets related to TFC tobacco control efforts were recorded. The data were provided by the program leader, and were collected from 2009–2013.

### 2.3. Data Analysis

Criteria for ranking progress was established and included the ability to establish a capable tobacco control team and accomplish the policy, program and media goals and objectives outlined in their proposals. The ranking strategy to measure progress included “exceeded goal”—met all goals and objectives outlined their action plan and expanded their programs to other sectors and/or adopted city-wide smoke-free policies; “met goal”—met all goals and objectives outlined in their action plan; and “made progress”—unable to meet all goals and objectives outlined in their action plan. Data collected from the bi-annual progress reports, site visits and conference calls were used to rank the grantees. Official policy documents were reviewed to validate policy adoption, inspection reports were analyzed to determine frequency, media campaigns were reviewed for content and media mentions were counted.

## 3. Results

The TFC grantees’ established tobacco control policies and implemented targeted programs and educational activities to raise public awareness, and their progress varied from exceeding expectations to making progress toward self-determined goals and objectives. The smoke free policy goals included creating 100% smoke free sectors: hospitals and community clinics (n = 3), government agencies (n = 7), schools (n = 5), enterprises (n = 2), restaurants and hotels (n = 1), and religious sites (n = 1). Four TFC grantees included program goals aimed at establishing 100% smoke free homes and families. Anshan, Changchun, Hangzhou, Kelamayi and Qingdao exceeded goal expectations; Changsha, Luoyang, Nanjing, Nanning, Suzhou, Tangshan, and Wuxi met their goals; and Bayannaoer, Dalian, Ningbo, Shanghai, and Yinchuan made progress toward their goals (Table 1).

Factors identified by TFC team leads in bi-annual progress report and during monthly conference calls and observed by the GHI-CTP during site visits as important to meeting or exceeding TFC goals and objectives included supportive government leaders, dedicated advocates and partners, non-smoking status of leaders and decision-makers (government and sector), parallel national Civilized or Healthy City initiative tobacco control goals, strong policies with implementation and enforcement strategies and media coverage. In addition, qualified, well-trained staff, adequate staffing levels to execute program activities, and defined action plans were also key to success. TFC grantees unable to meet their goals and objectives faced barriers and challenges such as the lack of support from leaders and decision-makers, frequent changes in leadership and staff, lack of engagement and support from key partners, and lack of adequately trained staff or insufficient staffing levels. Barriers to program success included any changes in leadership personnel from program inception, either in technical staff or in municipal officials, particularly when any of these individuals had not participated in making tobacco control a policy priority.

The TFC progress reports indicated that new or strengthened smoke free policies were adopted in their targeted city sectors in 2010–2012. Smoke free policy inspections were implemented, and the frequency varied from weekly to two times per year. Seven of the TFCs included the goal of adopting smoke free public places policies and two of these cities, Anshan and Kelamayi, successfully adopted city-wide smoke free policies in 2012. Their policies went into effect in early 2013 (Table 1).

In addition to policy adoptions, the TFC grantees conducted training on the newly adopted smoke free sector policies and included information on the harms of tobacco use and secondhand smoke to prepare the specific sector staff and enforcement agencies. They also assisted the sector sites with selection and placement of appropriate signage and outdoor smoking areas. Health education on the harms of tobacco use and secondhand smoke was also provided through a variety of channels including brochures and pamphlets, knowledge competitions, text messaging campaigns, websites and social media such as Twitter and Weibo. All of the TFC grantees engaged the media as a component of their TFC plan, including print, radio and news broadcasts and 1100 media mentions were reported. Eleven of the cities launched mass media campaigns. The various foci of the campaigns included education on the harms of tobacco use and secondhand smoke and new smoke free policies in the cities (Table 1).

Examples of cities that have made the most notable accomplishments include Kelamayi, Anshan, Qingdao, and Hangzhou. Kelamayi and Anshan initially focused their efforts on creating smoke free workplaces in large industries (petrochemical and steel respectively). The success of their smoke free workplace initiatives garnered the attention of the city government leaders, and the TFC grantees worked with their city’s legislative affairs office to draft smoke free public places policies that were adopted in late 2012. The Qingdao tobacco control team successfully led the efforts to create smoke free hospitals in their city. All 2147 of their hospitals adopted smoke free policies and they routinely monitor for compliance. As a result of their success, the Qingdao TFC team became sought-after tobacco control experts in their province. They are partnering with the local government to adopt a smoke free public places policy. Hangzhou’s tobacco control team partnered with hotel and restaurant managers and owners to pilot a 100% smoke free policy intervention that included training and technical support in 30 hospitality sites. They have expanded their model to include hotels and restaurants in the popular West Lake tourist district, and plan to continue the expansion throughout the city, as well as serving as a smoke free resource for the hospitality industry throughout China.

## 4. Discussion

As with World Bank Health VII, the TFC initiative demonstrated that policy change can occur and measures to change social norms can be successfully implemented in China cities. The TFC grantees made progress in establishing tobacco control teams and developing, implementing and evaluating plans to change social norms in their cities, with some experiencing more success than others. Important keys to establishing effective tobacco control programs included consistent political support, strong technical capabilities among the program team, knowledgeable and enthusiastic advocates and cooperative partners, comprehensive interventions, and policy inspections and appropriate enforcement. These keys are not unique to China, and have been identified as factors that make a difference by other global tobacco researchers [42,43].

Supportive government and organizational leaders were instrumental in the adoption and enforcement of smoke free policies. Although all TFC grantees were required to have government support at the mayoral level and organizational support to participate in the program, some of the city teams faced barriers and delays from government and organization leaders when they pushed for the adoption of smoke free policies. The ever-changing landscape of government and organizational leadership also played a role in some cities’ ability to achieve success.

Knowledgeable and capable tobacco control teams were also important to achieving TFC goals. While all of the TFC grantees were public health organizations, many of the program team members were new to the field of tobacco control and lacked the needed knowledge and skills required to successfully develop and implement effective tobacco control programs and evaluate their efforts. Concentrated trainings were provided to the TFC grantees on topics such as best and promising practices in tobacco control, tobacco control policies including policy development, implementation and enforcement, media and communication strategies, program planning, and developing and engaging partners. The TFC grantees were required to evaluate their program efforts, but early attempts at evaluation planning by the pilot cohort showed a lack of evaluation capacity. Frequent and consistent contact with the cities to monitor their progress and to be available to provide rapid response technical support was important to the success of the program. Monthly calls, frequent email exchanges, and site visits were integrated into the initiative to ensure that the TFC grantees were on target to meet their goals and objectives and that the cities technical support needs were met.

Engaging advocates and partner organizations in the city tobacco control efforts was shown to be an important component for success, yet some cities faced the challenges of identifying and engaging advocates, partners and collaborators in their work. Civil society has played a major role in tobacco control, particularly in Western countries, but this has not been the case in China. Even China’s non-government organizations (NGO) have significant ties to the government through funding, leadership and oversight. Also, while city health agencies have an important role to play in tobacco control program and policy efforts, some of the TFC grantees were unable to push forward policy and program efforts because of a lack of status in their city government hierarchy and influence with the city’s government leaders.

The TFC grantees were required to include policy, targeted program and media activities in their action plans and provided extensive training on internationally recognized best practices in tobacco control and ongoing technical assistance. Despite these efforts, there was a continued need to direct cities toward activities that have been shown to affect social norm change and away from relying solely on print materials, slogan buttons, and other less effective methods of communication. GHI-CTP was also concerned that some of the proposed interventions were too weak or too narrow in focus or reach to change social norms.

While policies, guidelines and recommendations are important first steps, they must be implemented and enforced to be effective in changing behaviors. Almost all of the participating cities reported that smoke free policies existed prior to their engagement but included loopholes such as allowing for smoking rooms, and were frequently not acknowledged or enforced. A lack of policy enforcement has also been recognized as a significant problem throughout China [6,12,13,14]. The grantees reported that the sector policies adopted during the project were in compliance with the FCTC Article 8, and inspection and enforcement strategies were included in the policy to ensure compliance. Analysis of the smoke free public places policies in Anshan and Kelamayi confirmed they were also in compliance with FCTC Article 8. Continued inspections will be needed to ensure that the policies are, in fact, enforced.

Capable technical teams and motivated partners and collaborators combined with support from their local government leaders are capable of implementing comprehensive tobacco control programs and strong policies aimed at changing the social norms of tobacco use.

## 5. Conclusions

Tobacco control, particularly in China, is complex, but the potential for significant public health impact is unparalleled. If men continue to smoke at current rates, and women initiate smoking as has been witnessed in other countries around the world, China will experience a burden of death and disease unseen in modern history. While policy changes at the central government level are necessary, national guidelines are unlikely to achieve their objectives without citizen support and acceptance at the city level. The current situation indicates that China’s ratification of the FCTC in 2006, the MOH national smoke-free hospital and smoke-free school policies and guidelines, and the prohibition of indoor smoking in public places in China have not been effective in changing smoking behaviors. Local interventions such as the adoption and enforcement of smoke-free policies, targeted program interventions, and media campaigns and health education are needed to complement national guidelines and to ensure effective tobacco control.

Cities have a critical role to play and the potential to change the tobacco control landscape in China. In fact, cities may be the driving force for social norm change related to tobacco use in China, and as such a key factor in both establishing governmental policies and ensuring that these policies are implemented and enforced.
